# *Coecobryasirindhornae* sp. n., the most highly troglomorphic Collembola in Southeast Asia (Collembola, Entomobryidae)

**DOI:** 10.3897/zookeys.824.31635

**Published:** 2019-02-12

**Authors:** Sopark Jantarit, Chutamas Satasook, Louis Deharveng

**Affiliations:** 1 Excellence Center for Biodiversity of Peninsular Thailand, Faculty of Science, Prince of Songkla University, Hat Yai, Songkhla, 90110, Thailand Prince of Songkla University Songkhla Thailand; 2 Princess Maha Chakri Sirindhorn Natural History Museum, Faculty of Science, Prince of Songkla University, Hat Yai, Songkhla, 90110, Thailand Prince of Songkla University Hat Yai Thailand; 3 Institut de Systématique, Evolution, Biodiversité (ISYEB) – UMR 7205 CNRS, MNHN, UPMC, EPHE, Museum national d’Histoire naturelle, Sorbonne Universités, 45 rue Buffon, CP50, F-75005 Paris, France Sorbonne Universités Paris France

**Keywords:** new species, peninsular Thailand, subterranean environment, taxonomy, troglomorphy

## Abstract

The most highly troglomorphic Collembola of Southeast Asia, *Coecobryasirindhornae***sp. n.**, is described from a cave in Satun province, Thai Peninsula. It is characterised by its large size, extremely elongated antennae, relatively long legs and furca, reduced macrochaetotaxy, very long and slender claw, pointed tenent hair, four sublobal hairs on outer maxillary lobe, and the absence of eyes and pigmentation. A checklist of Thai *Coecobrya* species and a key to the troglomorphic species of Thailand are provided. Troglomorphy and conservation of cave habitats in the area are discussed.

## Introduction

The genus *Coecobrya* Yosii, 1956 is characterised by polymacrochaetotic chaetotaxy, absence or reduced eye number, absence of or weak pigmentation, four antennal segments, falcate mucro with a basal spine, and absence of body scales, labral papillae, and dental spines (Deharveng 1990, Chen and Christiansen 1993, Zhang et al. 2009). So far, almost 60 species have been described worldwide (Bellinger et al. 1996–2019, Nilsai et al. 2017, Zhang et al. 2018). Among them, 30 species are cave dwellers. In Thailand, *Coecobrya* is widespread in both subterranean and epigean habitats throughout the country, with many forms still undescribed (Deharveng 1990, Jantarit et al. 2016). To date, fourteen species are recorded in the country (see checklist) of which twelve are cave-restricted. With regard to cave species, two morphological types can be recognised. The first type has short antennae and appendages, short and rather swollen claw and small size (0.9–1.75 mm). Species of this type are usually associated with eutrophic habitat, especially bat guano, and are never troglomorphic (see Deharveng 1990, Zhang et al. 2018). The second type has long appendages (antennae, and to a lesser degree legs and furca), slender claws and larger body size (1.72–2.82 mm; see Nilsai et al. 2017). Its species are always linked to oligotrophic habitats in the dark zone of caves with wet and moist environment (Nilsai et al. 2017). Cave *Coecobrya* of both types have all very narrow ranges in Thailand (Deharveng 1990, Jantarit et al. 2016, Nilsai et al. 2017, Zhang et al. 2018).

Troglomorphic features in Collembola are large body size, elongated appendages (antennae, and to a lesser degree legs and furca), multiplication of antennal chaetae, elongated and slender claw complex, pointed tenent hair, blindness and depigmentation (Christiansen 2012, Deharveng and Bedos 2018, Lukić et al. 2018). Troglomorphic Collembola are increasingly reported from the tropics, but the degree of troglomorphy in the species described so far is less strong than in temperate regions. Species which exhibit significant morphological adaptation to cave life in Southeast Asia include a single Neanuridae (*Coecolobaplumleyi* Deharveng, 1983), all others being Entomobryoidea of various genera: *Coecobrya*, *Cyphoderopsis* Carpenter, 1917, *Lepidonella* Yosii, 1960, *Pseudosinella* Schäffer, 1897, *Sinella* Brook, 1882, and *Troglopedetes* Absolon, 1907 (Deharveng 1987, Deharveng and Gers 1993, Deharveng and Bedos 2000, 2012, Jantarit et al. 2013, 2016, Nilsai et al. 2017, Deharveng et al. 2018). All are narrow range species. But in a broad regional context, data on their distribution are lacking throughout most karsts of the region (Deharveng and Bedos in press, Lukić in press).

In the present study, we describe an extremely troglomorphic new species of Collembola discovered in a cave in Satun province, Thailand. We discuss its level of troglomorphy, by far the highest for Collembola of continental Southeast Asia. A key to Thai troglomorphic species is provided.

## Materials and methods

We sampled at least 130 caves throughout Thailand to date. Collembola were collected by an aspirator or extracted on Berlese funnel from organic debris. The highly troglomorphic *Coecobrya* was found in a single cave, located in Satun province. Specimens were stored in 95% ethanol and were mounted on slides in Marc Andre II medium after clearing in Nesbitt solution. Morphological characters were examined using Leica DM1000 LED microscope with phase-contrast. Drawings were made using a drawing tube, and figures were improved with Illustrator CC (Adobe Inc). Specimens were brought alive to the laboratory, where photos were taken using a Leica M80 with Leica MC170 HD, and enhanced by LAS V4.12 software. Scanning Electron Micrographs were taken by Apreo SEM/FEI from the Scientific Equipment Center, Prince of Songkla University (Thailand).

**Abbreviations used in the description**:

Morphological structures:

**Ant.** antennal segment,

**Abd.** abdominal segment,

**psp** pseudopore(s),

**Th.** thoracic segment,

**Gr.** group,

**tita** tibiotarsus,

**mac** macrochaeta(e),

**mes** mesochaeta(e),

**mic** microchaeta(e),

**ms** S-microchaeta(e)/microsensillum(a),

**tric** trichobothrium(ia),

**s** ordinary S-chaeta(e)/sens

Institutions:

**BDCM**Biology department, Chiang Mai University, Chiang Mai, Thailand;

**BPBM**Bishop Museum, Honolulu, Hawaii;

**LEITT**Laboratoire d’Ecologie des Invertébrés Terrestres, Université Paul Sabatier, Toulouse, France (= Laboratoire de Zoologie, Université Paul Sabatier, Toulouse, France);

**MNHN**Museum national d’Histoire naturelle, Paris, France;

**NHM-PSU**Princess Maha Chakri Sirindhorn Natural History Museum, Prince of Songkla University, Songkhla, Thailand;

**NJAU** Department of Entomology, College of Plant Protection, Nanjing Agricultural University, China.

**Terminology**

Dorsal body chaetotaxy follows Szeptycki (1979) and Zhang et al. (2011). We use the notation of Zhang et al. (2016) for clypeal chaetotaxy, Fjellberg (1999) for labial palp. Dorsal chaetotaxy of head follows Jordana and Baquero (2005). Ventral chaetotaxy of head follows Chen and Christiansen (1993). Labial chaetae notation follows Gisin (1967), with the upper-case letter for ciliated and lower-case letter for smooth chaetae. The number of dorsal macrochaetae is given from Th. II‒Abd. IV. Symbols representing chaetal types used in the figures are as follows: large circle = macrochaeta; small circle = mesochaeta; cross = trichobothrium; and circle with a slash = pseudopore. Chaeta-to-chaeta homologies proposed here are indicative for several parts of the body where chaetae are known to be more or less variable in number or position.

## Taxonomy

### Class Collembola Lubbock, 1873

#### Order Entomobryomorpha Börner, 1913

##### Family Entomobryidae Tömösváry, 1882

###### Subfamily Entomobryinae Schäffer, 1896

####### Genus *Coecobrya* Yosii, 1956

######## Coecobrya sirindhornae sp. n.

Taxon classificationAnimaliaEntomobryomorphaEntomobryidae

http://zoobank.org/E4EADEEB-F274-4CAE-9ABB-40FF14934965

**Figure 1.**
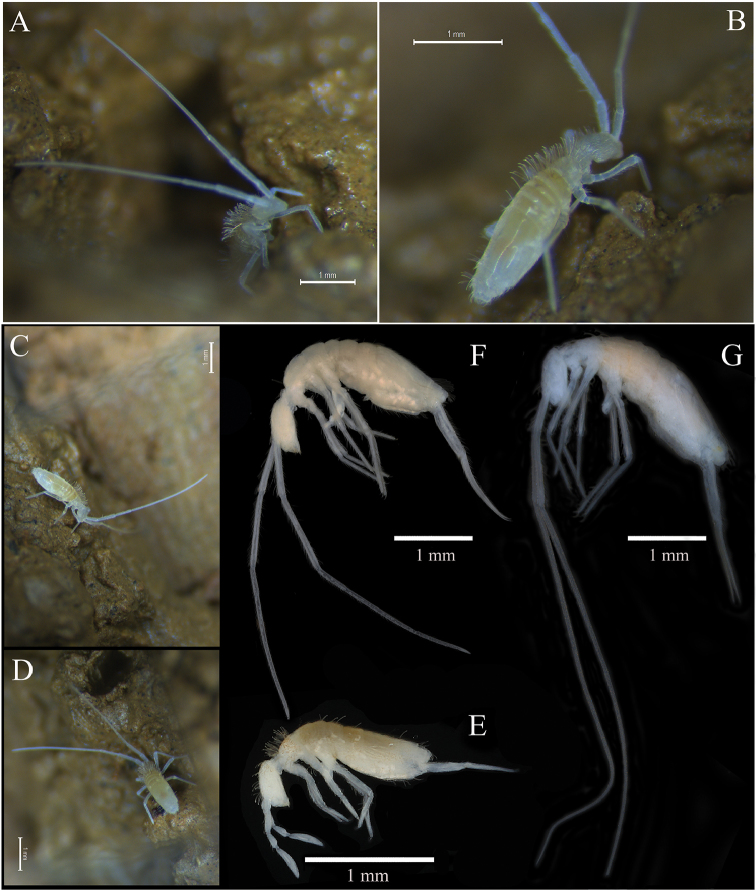
*Coecobryasirindhornae* sp. n. **A–D** Habitus **E–F** Two morphological types of cave *Coecobrya* in Thailand **E***Coecobryaphanthuratensis* Zhang & Jantarit, 2018; normal form with short antennae, appendages and small size **F***Coecobryapolychaeta* Zhang & Nilsai, 2017; troglomorphic form with long antennae and appendages with large body size and **G***Coecobryasirindhornae* sp. n., highly troglomorphic characters with extremely long antennae and appendages and also large body size.

**Figure 2.**
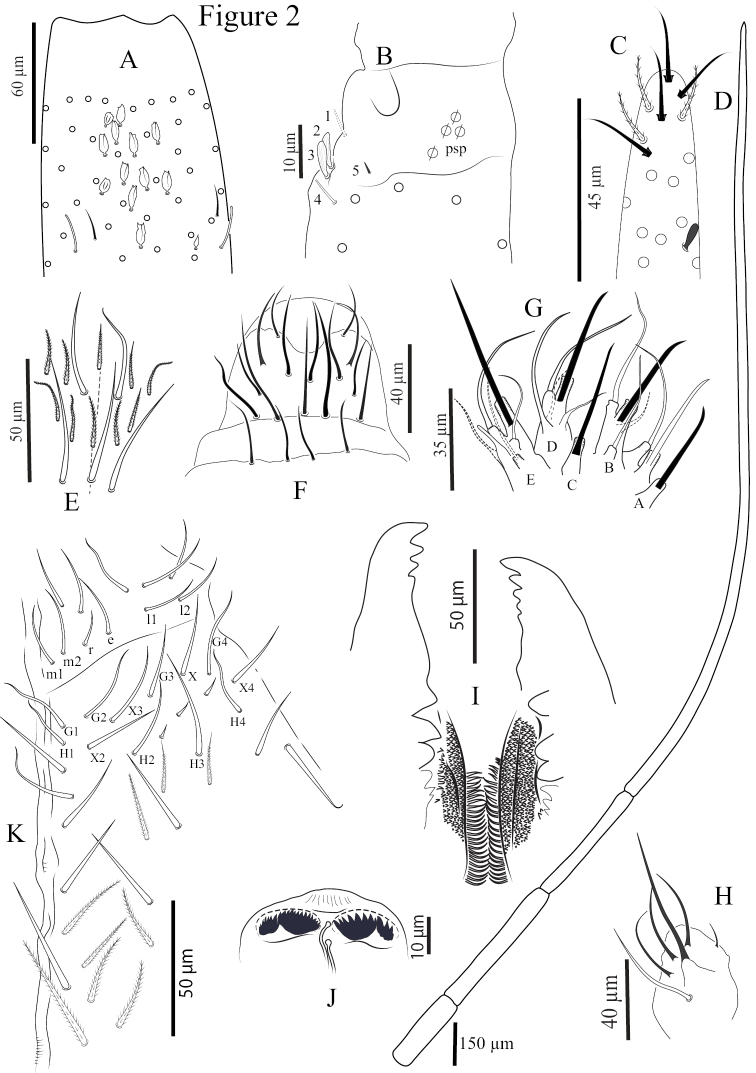
*Coecobryasirindhornae* sp. n. continued. **A** Distal part of Ant. II dorsally of left antenna **B**Ant. III organ of left side **C** Distal part of Ant. IV with subapical organite **D** Ratio of antennal length **E** Clypeal chaetae **F** Prelabral and labral chaetae **G** Labial palp **H** Outer maxillary lobe **I** Mandibles **J** Ventro-distal complex of labrum **K** Chaetae of labial basis and ventral chaetotaxy of head.

**Figure 3.**
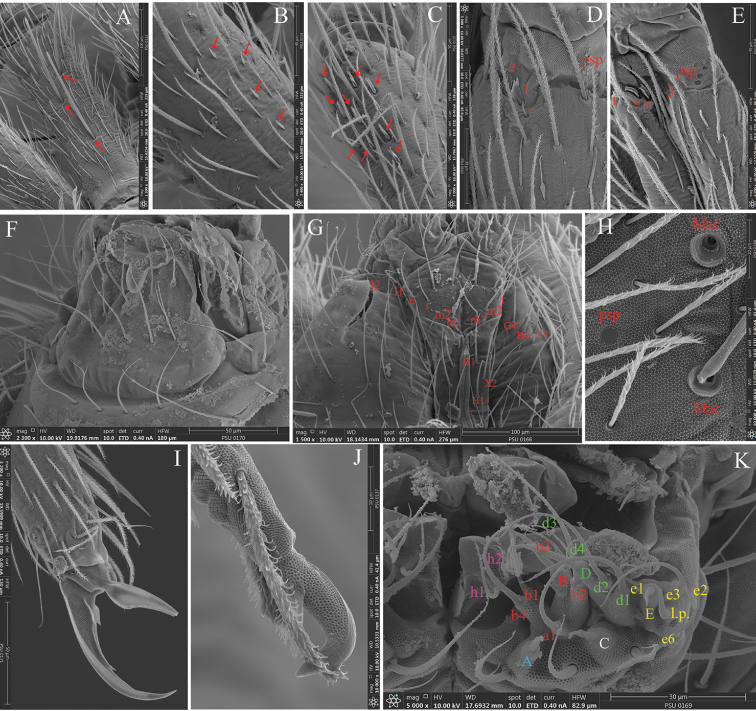
*Coecobryasirindhornae* sp. n. continued with SEM. **A**Ant. I dorsally with three mac (arrows) **B**Ant. I latero-dorsally with a row of spear-like chaetae (arrows) **C** Distal part of Ant. II dorsally with a group of paddle-like chaetae (arrows) **D–E**Ant. III organ of right side **F** Labral chaetae and maxillary outer lobe **G** Ventral chaetotaxy of head **H** Pseudopore and mac on Th. III **I** Claw III morphology **J** Mucro **K** Labial palp with its notation after Fjellberg (1999).

**Figure 4.**
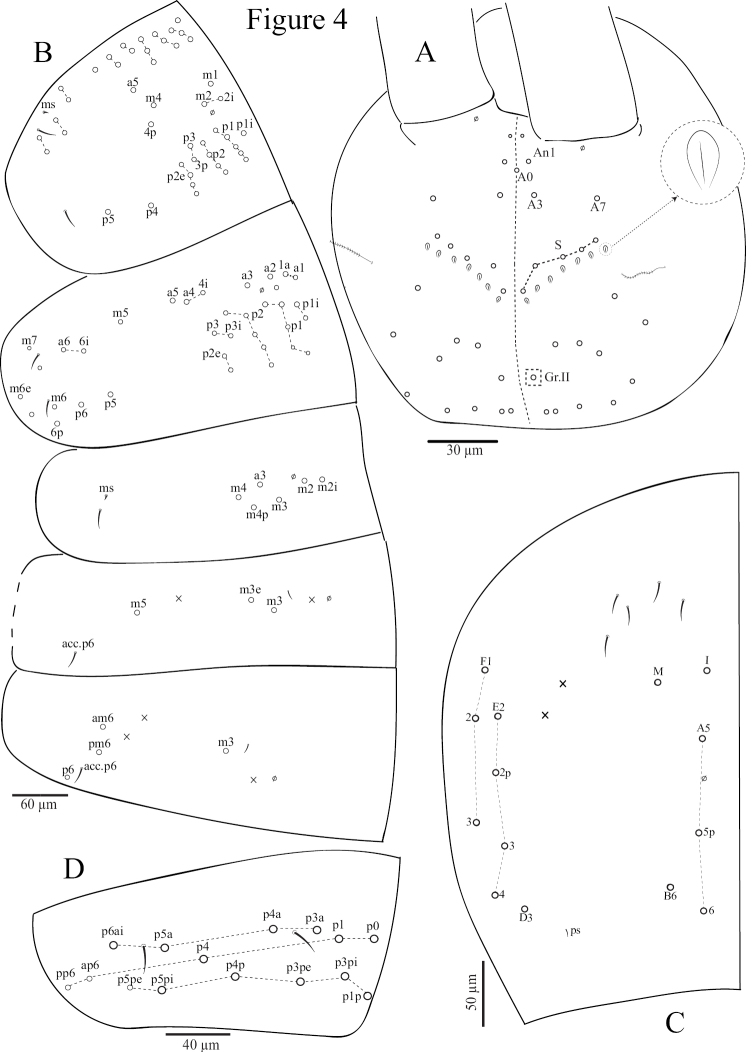
*Coecobryasirindhornae* sp. n. continued. **A** Chaetotaxy of dorsal head **B** Chaetotaxy of dorsal Th. II-Abd. III **C** Chaetotaxy of dorsal Abd. IV **D** Chaetotaxy of dorsal Abd. IV.

**Figure 5.**
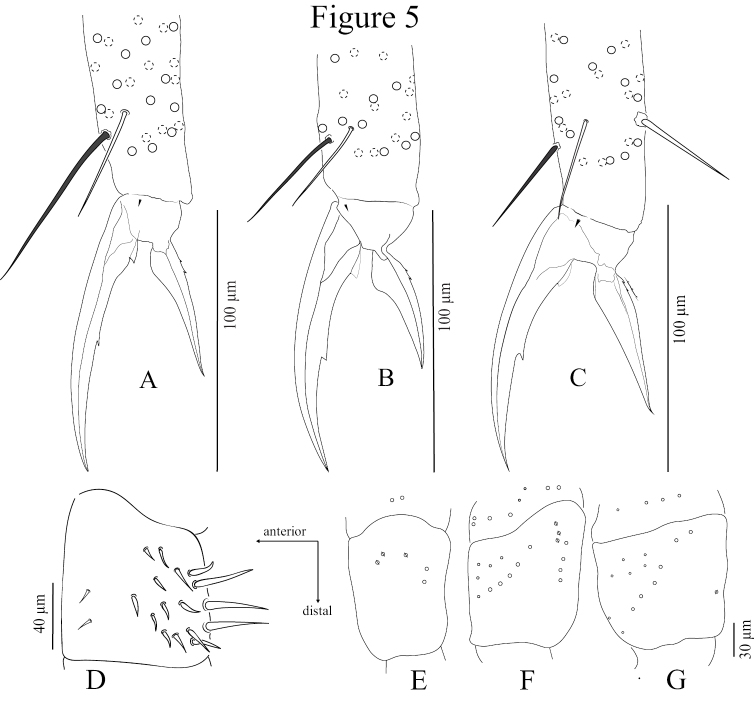
*Coecobryasirindhornae* sp. n. continued. **A** Distal part of tita I and claw complex **B** Distal part of tita II and claw complex **C** Distal part of tita III and claw complex **D** Trochanteral organ **E** Pseudopores and chaetae sockets of coxae I–III (left to right).

**Figure 6.**
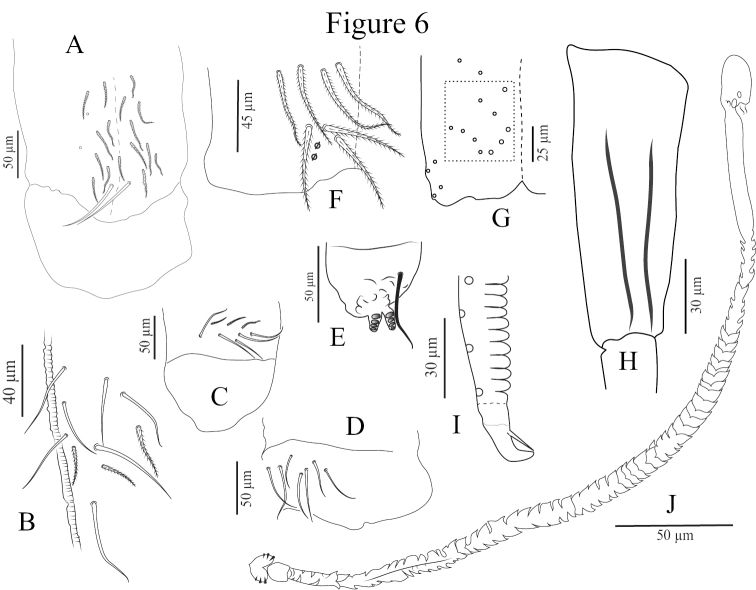
*Coecobryasirindhornae* sp. n. continued. **A** Posterior side of ventral tube **B** Anterior side of ventral tube **C, D** Lateral flap **E** Tenaculum **F** Manubrium plaque **G** Distal part of manubrium ventrally **H** Manubrium with two thin, straight longitudinal structures inside **I** Mucro **J** Spermathecal duct.

**Figure 7.**
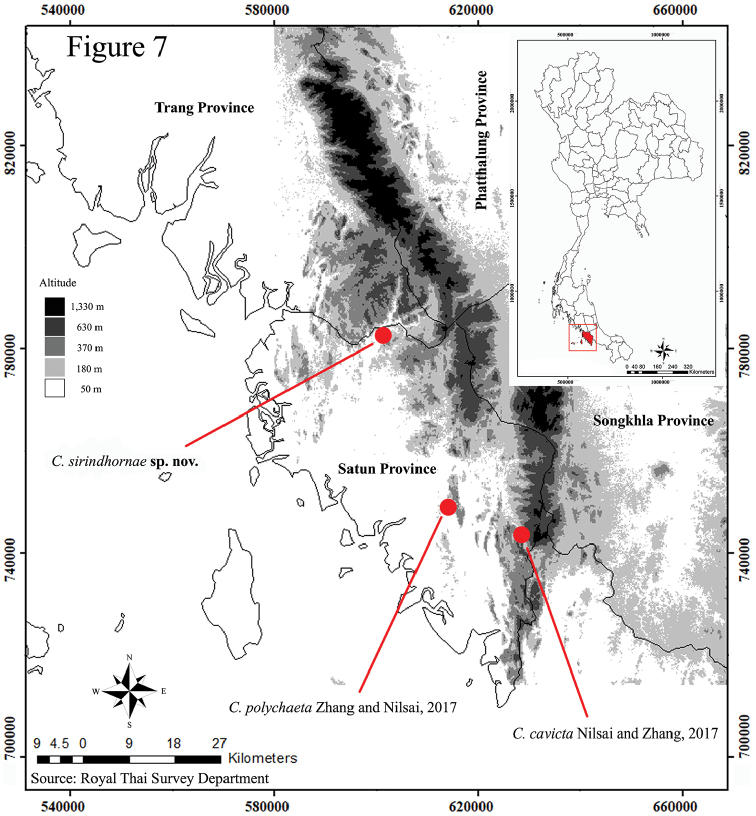
Distribution of three troglomorphic *Coecobrya* species in Satun caves, all located in lowland areas.

Figs 1 , 2 , 3 , 4 , 5 , 6 , 7

######### Type material.

***Holotype***: male on slide, Thailand: Satun province: Manang district, Tham Rusri, altitude 58 m, nine specimens (one male, one female and three subadults in slides, three in ethanol), dark zone of cave, by aspirator, S Jantarit and A Nilsai leg. (sample # THA_SJ_STN09), 30/04/2016 (A Nilsai), six specimens (three subadults in slides, three in ethanol); 03/05/2016 (S Jantarit and A Nilsai), five specimens in ethanol; 25/07/2017 (S Jantarit and A Nilsai), three specimens in ethanol; 17/03/2018 (S Jantarit and A Nilsai), three specimens in ethanol. Holotype and 13 paratypes in slides deposited in NHM-PSU. Two paratypes in alcohol in MNHN. Three paratypes on slides and three in alcohol in NJAU. Tham = cave (in Thai).

######### Description.

*Habitus* (Fig. 1A‒D, G). Medium size Entomobryidae. Body length up to 2.6 mm (holotype 2.1 mm). No scales. Eyes absent. Colour: pale yellow to whitish in alcohol, without pigments. Four antennal segments (sometimes Ant. III and IV fused together). Body slender with very long antennae and moderately elongate legs and furca. Body not bent nor humped at level of Th. II. Th. II slightly longer than Th. III; Abd. IV 3‒4 times as long as Abd. III.

*Pseudopores* (Figs 2B, 3D‒E, 3H, 4A‒C, 5E‒G, 6F). Pseudopores present as round flat disks, smaller than mac sockets (Figs 3H, 4A‒C), except for the coxae and manubrium where psp are as large as mac sockets, present on various parts of the body: antennae, head, tergites, coxae and manubrium. On antennae, psp located ventro-apically between the tip of antennal segments and the chaetae of the apical row, or just below apical row of chaetae (two on Ant. I, 2‒3 on Ant. II, and 4‒7 on Ant. III) (Figs 2B, 3D‒E). On head, 1‒(2) psp located externally on each peri-antennal area (Fig. 4A). On tergites, 1+1 psp close to the axis from Th. II to Abd. IV (Fig. 4B, C). Coxae I, II, and III with 2‒ (3), 2‒ (3), and 1‒2 psp respectively, located close to longitudinal rows of chaetae (Fig. 5E‒G). On manubrium, 2+2 dorso-apical ones (Fig. 6F).

*Mouthparts and ventral head chaetotaxy* (Figs 2E‒J, 3F, 3K). Clypeal area with three long, smooth prefrontal and 6‒10 ciliated and two long smooth facial chaetae (Fig. 2E), sometimes asymmetric arrangement. Distal border of the apical non-granulated area of the labrum with a relatively narrow median U-form intrusion into the granulated area dorsally; apical edge not adorned with spines (Figs 2F, 3F). Ventro-distal complex of labrum well differentiated, asymmetrical, with 1+1 distal combs of 13‒21 minute teeth on the left side and 10‒11 strong and larger teeth on the right side, and an axial pair of long sinuous tubules, round apically (Fig. 2J). Prelabral and labral chaetae 4/5, 5, 4, all thin and smooth; three median chaetae of the first and second rows longer and slightly larger than those of the distal and proximal rows (35‒45 vs, 30 µm)(Figs 2F, 3F). Maxillary outer lobe with one papillate chaeta, one basal chaeta, and four sublobal hairs of which the upper one is three times shorter than the others (Figs 2H, 3F). Labium and ventral head (Figs 2G, K, 3G, K). Labial palp strongly modified for the genus, with 0, 5, 0, 4, 4 guards for papillae A‒E, like that described by Fjellberg (1999) for Entomobryidae or by Xu and Zhang (2015) for *Coecobrya*. Lateral process of labial palp subcylindrical, as thick as normal chaetae, with tip slightly beyond apex of labial papilla (Figs 2G, 3K). Five smooth and acuminate proximal chaetae. Chaetae of labial basis all smooth (m_1_m_2_rel_1_l_2_); chaetae m_2_ slightly larger and longer than m_1,_ chaetae m_1_, e and l_1_ subequal, r thin and shortest, and l_2_ longest (Figs 2K, 3G). One short and smooth chaeta present in one individual between m_2_ and r, other two chaetae of the submentum smooth and acuminate, of similar size. Postlabial chaetae X_2_, X_3_, X and X_4_ smooth, long and acuminate, X_1_ absent; 2‒5 smooth and minute chaetae between H_2_ and H_3_. On each side of linea ventralis, 7‒9 smooth and 3‒7 ciliate chaetae, the anterior 6 always long, smooth and acuminate, the posterior ones either smooth or ciliated (Figs 2K, 3G). Mandible apex strong, asymmetrical (left with four teeth, right with five teeth); molar plate with three strong pointed basal tooth, and 3‒(5) smaller inner distal teeth, identical in both mandibles (Fig. 2I). Maxilla capitulum with a three-toothed claw and several stout ciliated lamellae; lamella 2 large and broad, lamella 3 well developed; several other lamellae present.

*Antennae* (Figs 1A‒D, 1G, 2A‒D, 3A‒E). Antennae extremely long, approximately 8.0–12.3 times as long as cephalic diagonal and 2.0‒2.2 times longer than (head + body). Antennal segment ratio as I : II : III : IV = 1 : 1.3–1.9 : 1.6–2.0 : 8.1–11.3 (N = 5). Antennal segments not subdivided nor annulated. At least three specimens with asymmetrical antennae, one with four segments and the other one slightly shortened with three segments; two specimens with three antennal segments of both sides. Antennal chaetal types not analysed in detail. Ant. I dorsally with three mac (Fig. 3A) and a row of 2‒4 spear-like chaetae latero-dorsally (Fig. 3B). Ant. I ventrally with many smooth spiny mic of various sizes in its basal part, many subcylindrical, hyaline sens in its middle to apical part, and many long smooth straight chaetae. Ant. II dorsally with 10‒12 paddle-like chaetae (sensu Nilsai et al. 2017) in its distal part (Figs 2A, 3C). Ant. III organ with five sens; sens one and four subequal, hyaline; sens five acuminate, dark and shorter; sens two and three swollen resting in shallow groove (Figs 2B, 3D‒E), not clearly seen in most specimens. Ant. IV very long, not subdivided, without apical bulb (Fig. 2C‒D). Subapical organite not distinctly knobbed, swollen, slightly enlarged apically, inserted dorsally at 35‒45 µm from the tip (Fig. 2C).

*Dorsal head chaetotaxy* (Fig. 4A). Dorsal cephalic chaetotaxy with one antennal (An), without median (M) and five sutural (S) mac; Gr. II with only one mac; A_0_ as mes; 7+7 scale-like structures (2‒3 µm) present below sutural mac, probably inside the integument; a pair of short cephalic trichobothria, external and close to the middle of the head (Fig. 4A).

*Tergites* (Fig. 4B–D). Th. II with three (m1, m2, m2i) medio-medial, two (m4, m4p) medio-sublateral and 15‒18 posterior mac; 1+1 ms and 1+1 sens antero-laterally. Th. III with 32‒35 mac; a1a as mac. 2+2 sens laterally. Abd. I with six (a3, m2–4, m2i, m4p) mac. 1+1 ms and 1+1 sens laterally. Abd. II with two (m3, m3e) central and one (m5) lateral mac. 2+2 tric without modified chaetae, 1+1 sens laterally and 1+1 mic near internal tric. Abd. III with one (m3) central and three (am6, pm6, p6) lateral mac. 3+3 tric not surrounded by modified chaetae, 1+1 sens laterally,1+1 mic near m3, ms not seen (Fig. 4B). Abd. IV with six central mac (I, M, A5–6, A5p, B6) and eight (D3, E2–4, E2p, F1–3) lateral mac, 2+2 tric and approx. 5‒7 long S-like chaetae anteriorly, without modified chaetae (Fig. 4C). Abd. V with 13‒15 mac and 2+2 sens (Fig. 4D). Abd. VI not analysed. S-chaetae formula from Th. II to Abd. V: 2+ms, 2/1+ms, 2, 2+ms, 1+ ≈5‒7, 2; as sens not seen and ps on Abd. IV 1/4 as long as S-like chaetae (Fig. 4C).

*Legs* (Figs 3I, 5A‒G) long; tita of leg III slightly longer than tita of legs I and II. Legs devoid of scales, covered with ordinary ciliated chaetae of various lengths, mic not seen. Coxa of leg I with three proximal psp and two chaetae posteriorly; coxa of leg II with 8–10 chaetae (5–6 mac) in anterior row, 3–4 chaetae (mac) in posterior row and 2–3 proximal psp in between; coxa of leg III with 13+15 chaetae (6–7 mac) in anterior row, and one proximal psp posteriorly. Trochanteral organ with 12‒18 smooth, straight, unequal spine-like chaetae (Fig. 5D). The distal whorl of tita with 10‒12 subequal ciliated mes, irregularly arranged, and a thin, acuminate, smooth dorso-apical tenent hair. Tenent hair of tita I longer (50‒65 µm) than that of tita II and III (30‒40 µm) (Fig. 5A‒C). Tita I-III with one smooth, thin and long chaetae close to tenent hair (25‒40 µm, N = 5) (Figs 3I, 5A‒C). Ventro-distal smooth chaeta of tita III thick, erected, pointed, rather short (35‒40 µm, N = 5). Pretarsal mic minute (2.5‒3.0 µm). Claw slender and elongated; claw I and II subequal (60‒98 µm long, 7‒12 µm wide at basis), claw III slightly longer (80‒100 µm long, 15‒17 µm wide at basis) (N = 5). All claw with one strong inner tooth at 50‒55% and a pair of basal inner teeth at approx. 22‒25% of inner edge from basis. Unguiculus approx. 3/5 as long as inner edge of claw, slightly swollen baso-internally, pointed apically, devoid of inner tooth, not truncated, with 2‒3 minute outer teeth, often inconspicuous, at 1/3 of its length (Figs 3I, 5A‒C).

*Ventral tube* (Fig. 6A‒D). Ventral tube four times longer than wide. Lateral flaps with 7‒8+7‒8 smooth chaetae (sometimes with 5+5 ciliated and 3+3 smooth) (Fig. 6C‒D). Anteriorly with 10+10 large chaetae, 3+3 ciliated and 7+7 smooth, two of them larger (Fig. 6B); posteriorly with 20‒30 mes, all serrated, arranged roughly asymmetrically, with 1+1 smooth, straight, distal mac close together (Fig. 6A).

*Furcal complex* (Figs 3J, 6E‒I). Tenaculum with four large teeth of decreasing size from the basal to the distal one of each ramus, on a prominent, irregular body, with a postero-basal strong serrated chaeta bent distally (Fig. 6E). Mucrodens 1.25‒1.60 times longer than manubrium. Furcula without smooth chaetae. Manubrium with a dense cover of ciliated chaetae both dorsally and ventrally. Manubrial plaque with two pseudopores and three ciliate chaetae (Fig. 6F). Distal part of manubrium ventrally with 8‒10 ciliate chaetae on each side, four of them mac (Fig. 6G). Inside the manubrium, two thin, straight longitudinal structure running on ¾ of manubrium length from its apex like in *Lepidonelladoveri* (Carpenter, 1933) (after Deharveng et al. 2018) (Fig. 6H). Dens without spines, annulated and covered with ciliated chaetae on both sides. Distal smooth part of dens slightly shorter than mucro. Mucro strong and falcate, basal spine long, nearly reaching the tip of the mucronal tooth (Figs 3J, 6I).

*Genital plate* (Fig. 6J). Male genital plate with 3+3 genital mic, acuminate circumgenital mes not clearly seen, without modified chaetae. Spermathecal duct elongated and annulated (Fig. 6J). Female genital plate not clearly seen.

######### Ecology.

*Coecobryasirindhornae* sp. n. is restricted to the dark zone of the cave where it has been found, in the oligotrophic environment of a small chamber, on muddy ground and wet rock walls. The chamber is connected to a narrow steep hole. Small puddles are present in the chamber and water is dripping from the ceiling. Muddy ground surface is flooded during rainy season. Some individuals were found feeding on a cricket corpse. They were quick jumping and moved rapidly. The species is found only in that chamber where humidity is at saturation, and temperature is constant (23‒24 degrees Celsius). The population seems rather limited (only 26 specimens were collected from five attempts, each time one hour collecting by 2 people). Small (young) individuals were less numerous and not collected.

######### Etymology.

This species is named to honour Her Royal Highness Princess Maha Chakri Sirindhorn, who is passionately interested in natural history and plays an important role in promoting the conservation of biodiversity and the environment of Thailand.

######### Remarks.

*Coecobryasirindhornae* sp. n. differs at first from all other species of the genus by its highly troglomorphic characters. Diagnostic morphological characters of the new species and related troglomorphic *Coecobrya* are listed in Table 1. *Coecobryasirindhornae* sp. n. is well characterised by the combination of large body size, extremely long antennae, all labial chaetae smooth, elongated lateral process of labial palp and four sublobal hairs on maxillary outer lobe, very elongated and slender claw, presence of 2‒3 minute teeth on outer edge on unguiculus, less chaetae on ventral tube and both sides of distal part of manubrium, and reduced dorsal chaetotaxy of both head and tergites. Head is without M row and with internal scale-like structures below the sutural mac. We have been unable to detect the third pair of sens on Abd. V, but we do not consider this absence as diagnostic as it would be a very unusual feature for a *Coecobrya*, and it is often difficult to observe or fallen down. Antennae of the new species are the longest known in the genus, longer than *C.nupa* Christiansen & Bellinger, 1992 from Hawaii, previously the species with the longest antennae; and *C.polychaeta* Zhang & Nilsai, 2017 and *C.chumphonensis* Zhang & Nilsai, 2017 (both in Nilsai et al. 2017) from Thai peninsula (Table 1). According to the three troglomorphic species of Thailand, *C.polychaeta* comes near to *C.sirindhornae* sp. n. in body length, colour, clypeus chaetae, elongated lateral process of labial palp and number of sublobal hairs on maxillary outer lobe, but can be clearly differentiated from it by the characters listed in Table 1. The other two taxa from Thailand are not close to the new species. *Coecobryanupa*, the first report of highly troglomorphic species in the genus, differs from the new species mainly in shorter antennae length, labial basis chaetotaxy, claw morphology and mucronal spine exceeding the tip of the apical tooth (Table 1). The distribution map of *C.sirindhornae* sp. n. and the other two troglomorphic *Coecobrya* discovered in Satun province is shown in Figure 7.

**Table 1.** Comparison of troglomorphic *Coecobrya*: *C.chumphonensis* Zhang & Nilsai, 2017, *C.cavicta* Nilsai & Zhang, 2017, *C.polychaeta* Zhang & Nilsai, 2017, *C.sirindhornae* sp. n., and *C.nupa* Christiansen & Bellinger, 1992. Key: c = ciliated chaetae, s = smooth chaetae, ? = not given in literature description. Characters indicated in parentheses are rarely observed.

Characters	* C. chumphonensis *	* C. cavicta *	* C. polychaeta *	*C.sirindhornae* sp. n.	* C. nupa *
Body length	up to 2.82 mm	1.72 mm	up to 2.58 mm	up to 2.6 mm	2.0 mm
Ant. /head	3.70–4.48	2.67	5.91–7.12	8.0‒12.3	6.5
Long smooth straight chaetae on antennae	absent	present	absent	present	?
Number of paddle-like chaetae on Ant. II	2‒4	?	1	10‒12	?
Clypeus					
prefrontal	3s	?	3s	3s	?
facial	8s	?	2s7‒10c	2s7‒10c	?
Dorsal head					
An	2	4	4	1	?
A0	mac	mac	mic	mes	?
M	4	3	3	0	?
Gr. II	6(7)	4	3	1	?
Sublobal hairs on maxillary outer lobe	3	3	4	4	3
Lateral process of labial palp	short	short	long	long	short
Labial chaetae	mRel_1_l_2_	mrel_1_l_2_	M_1_m_2_rel_1_l_2_	m_1_m_2_rel_1_l_2_	M_1_m_2_rel_1_l_2_
Postlabial chaeta X	minute	minute	normal	normal	?
Chaetae along cephalic groove	4s5‒7c	3‒4s7‒8c	6‒7s5‒10c	7‒9s3‒7c	?
Chaetotaxy of Th. II					
medio-medial mac	4–6	3	7(6)	3	2
medio-sublateral mac	3	4	3	2	?
Posterior macTh. II	25–32	30–31	29–40	15–18	?
Mac on Th. III	32–35	35	35–43	32–35	15?
Mac on Abd. I	6–7	6–7	8–9	6	4
Central macAbd. II	3	3	4(3)	2	2
Chaetotaxy of Abd. III					
central mac	2	1	1	1	1
lateral mac	3	3	3	3	2?
ms	not seen	not seen	present	not seen	?
Chaetotaxy of Abd. IV					
central mac	7	7‒9	6	6	4
lateral mac	10–12	11	9	8	?
Tenent hair	usually pointed	pointed	pointed	pointed	pointed
Ungual inner teeth	3	2	3	3	3
Unguiculus outer edge	serrate	serrate	serrate	2–3 teeth	?
Ventral tube					
anterior face	9‒12	?	12	10	?
posterior face	13	?	20–31	20–30	7
lateral flap	7(10)	?	9–12	7–8s(5c)	6
Smooth chaetae trochanteral organ	12–22	15–16	15–25	12–18	16
Chaetae on manubrial plaque	4‒7	4	4‒10	3	5
Chaetae on ventro-distal part of manubrium	11–15c	13c	15–25c	8–10c	?
Mucronal spine	nearly reaching mucronal apex	nearly reaching mucronal apex	nearly reaching mucronal apex	nearly reaching mucronal apex	beyond mucronal apex
Locality	Chumphon, Thailand	Satun, Thailand	Satun, Thailand	Satun, Thailand	Maui, Hawaii

####### Checklist of Thai species of *Coecobrya*

Jantarit et al. (2016) listed only three species of genus *Coecobrya* and erroneously did not include *C.lanna* Zhang, Deharveng & Chen, 2009 in the checklist of Collembola of Thailand. Recently, ten newly discovered species were described by Nilsai et al. (2017) and Zhang et al. (2018). *Coecobrya* species are listed here by including the valid names, author(s) and year of publication, source(s), type deposition, altitude, coordinates (if available), ecological data, and distribution. Records are updated to 10/01/2019.

**Coecobryacf.hoefti (Schäffer, 1896)**

**Sources.**Yosii 1961, Takeda 1981, Deharveng 1986, Deharveng et al. 1989, Rojanavongse et al. unpublished report, Jantarit et al. 2016.

**Altitude.** > 2,000 m a.s.l. in Doi Inthanon, Chiang Mai province (Yosii 1961); 800 m a.s.l. in Chaiyaphum province (Takeda 1981) (given as Khon Kaen province in Jantarit et al. 2016; Khon Kaen experimental farm is actually located in Chaiyaphum).

**Habitat.** No ecological data in Chiang Mai province; soil sample in mixed dry deciduous forest and deforested area in Chaiyaphum province.

**Distribution.** Doi Inthanon, Chiang Mai province, northern Thailand and Khon San district, Chaiyaphum province, northeastern Thailand.

**Distribution outside Thailand**. Europe.

***Coecobryaguanophila* Deharveng, 1990**

**Sources.**Deharveng 1990, Bedos 1994, Deharveng and Bedos 2001, Rojanavongse et al. unpublished report, Jantarit et al. 2016.

**Type deposition.**BDCM, BPBM, LEITT, MNHN.

**Altitude.** 458 m a.s.l.

**Coordinates.** 19°23.6386'N; 98°55.6864'E

**Habitat.** Dark zone of cave (Tham Chiang Dao) on bat guano.

**Distribution.** Chiang Dao district, Chiang Mai province, northern Thailand (only known from the type locality).

***Coecobryasimilis* Deharveng, 1990**

**Sources.**Deharveng 1990, Bedos 1994, Rojanavongse et al. unpublished report, Jantarit et al. 2016.

**Type deposition**: BDCM, BPBM, LEITT, MNHN.

**Altitude.** 500 m a.s.l.

**Coordinates.** around Tham Chiang Dao (19°23.6386'N; 98°55.6864'E)

**Habitat.** Near the entrance of Tham Chiang Dao, litter and soil.

**Distribution.** Chiang Dao district, Chiang Mai province, northern Thailand (only known from the type locality).

***Coecobryalanna* Zhang, Deharveng & Chen, 2009**

**Sources.**Zhang et al. 2009, Cipola and Bellini 2016, Nilsai et al. 2017.

**Type deposition.** MNHM and NJAU.

**Altitude.** 1,720 m a.s.l.

**Coordinates.** 19°23.5213'N; 98°52.4899'E

**Habitat.** Forest litter at Doi Chiang Dao.

**Distribution.** Chiang Dao district, Chiang Mai province, northern Thailand (only known from the type locality).

***Coecobryacavicta* Nilsai & Zhang, 2017**

**Source.**Nilsai et al. 2017.

**Type deposition.**NHM-PSU.

**Altitude.** 115 m a.s.l.

**Coordinates.** 6°43.5816'N; 100°9.7494'E

**Habitat.** Dark zone of cave (Tham Ton Din) in wet and humid environment, near a stream bank, without bat guano.

**Distribution.** Khuan Don district, Satun province, southern Thailand (only known from the type locality).

***Coecobryachumphonensis* Zhang & Nilsai, 2017**

**Source.**Nilsai et al. 2017.

**Type deposition.**NHM-PSU and NJAU.

**Altitude.** 70 m a.s.l.

**Coordinates.** 10°26.7841'N; 99°2.1018'E

**Habitat.** Dark zone of cave (Tham Chang Puak) on wet ground and rocks near a puddle; wet and humid environment, in an oligotrophic habitat without bat guano.

**Distribution.** Mueang district, Chumphon province, southern Thailand (only known from the type locality).

***Coecobryapolychaeta* Zhang & Nilsai, 2017**

**Source.**Nilsai et al. 2017.

**Type deposition.**NHM-PSU and NJAU.

**Altitude.** 23 m a.s.l.

**Coordinates.** 6°46.5246'N; 100°2.4966'E

**Habitat.** Dark zone of cave (Tham Phraya Bangsa) in a small chamber of muddy ground, wet and humid environment, in an oligotrophic habitat without bat guano.

**Distribution.** Mueang district, Satun province, southern Thailand (only known from the type locality).

**Coecobryacf.polychaeta Zhang & Nilsai, 2017**

**Source.**Nilsai et al. 2017.

**Altitude.** 115 m a.s.l.

**Coordinates.** 6°43.5816'N; 100°9.7494'E

**Habitat.** Dark zone of cave (Tham Ton Din) along a stream bank, on stalagmites, muddy and clay substrate, gravels and rocks in wet and humid environment, without bat guano.

**Distribution.** Khuan Don district, Satun province, southern Thailand (only known from this cave).

***Coecobryadonyoa* Zhang & Jantarit, 2018**

**Source.**Zhang et al. 2018.

**Type deposition.**NHM-PSU and NJAU.

**Altitude.** 65 m a.s.l.

**Coordinates.** 9°54.238'N; 99°2.685'E

**Habitat**. Dark zone of cave (Tham Don Yoa) on bat guano.

**Distribution**. Lang Suan district, Chumphon province, southern Thailand (only known from the type locality).

***Coecobryakhaopaela* Zhang & Jantarit, 2018**

**Source.**Zhang et al. 2018.

**Type deposition.**NHM-PSU and NJAU.

**Altitude.** 300 m a.s.l.

**Coordinates.** 9°33.5599'N; 98°58.9364'E

**Habitat.** Dark zone of cave (Tham Khao Paela) on bat guano.

**Distribution.** Tha Chana district, Surat Thani province, southern Thailand (only known from the type locality).

***Coecobryakhromwanaramica* Zhang, 2018**

**Source.**Zhang et al. 2018.

**Type deposition.**NHM-PSU and NJAU.

**Altitude.** 77 m a.s.l.

**Coordinates.** 8°46.194'N; 99°22.106'E

**Habitat.** Twilight to dark zone of a rather dry cave (Tham Khromwanaram) on bat guano.

**Distribution.** Ban Na San district, Surat Thani province, southern Thailand (only known from the type locality).

***Coecobryaphanthuratensis* Zhang & Jantarit, 2018**

**Source.**Zhang et al. 2018.

**Type deposition.**NHM-PSU and NJAU.

**Altitude.** 82 m a.s.l.

**Coordinates.** 8°54.028'N; 98°31.498'E

**Habitat.** Twilight to dark zone of cave (Tham Phanthurat) on bat guano.

**Distribution.** Phanom district, Surat Thani province, southern Thailand (only known from the type locality).

***Coecobryapromdami* Zhang & Jantarit, 2018**

**Source.**Zhang et al. 2018.

**Type deposition.**NHM-PSU and NJAU.

**Altitude.** 78 m a.s.l.

**Coordinates.** 9°12.293'N; 99°46.47'E

**Habitat.** Dark zone of humid cave (Tham Khao Wang Thong) on bat guano.

**Distribution.** Khanom district, Nakhon Si Thammarat province, southern Thailand (only known from the type locality).

***Coecobryaranongica* Nilsai & Zhang, 2018**

**Source.**Zhang et al. 2018.

**Type deposition.**NHM-PSU and NJAU.

**Altitude.** 52 m a.s.l.

**Coordinates.** 10°19.5745'N; 98°45.9012'E

**Habitat.** Twilight to dark zone of cave (Tham Phra Khayang) on bat guano.

**Distribution.** Kra Buri district, Ranong province, southern Thailand (only known from the type locality).

***Coecobryaspecusincola* Zhang & Nilsai, 2018.**

**Source.**Zhang et al. 2018.

**Type deposition.**NHM-PSU and NJAU.

**Altitude.** 160 m a.s.l.

**Coordinates.** 8°59.996'N; 99°46.692'E

**Habitat.** Dark zone of cave (Tham Khao Phab Pha) on scattered bat guano.

**Distribution.** Sichon district, Nakhon Si Thammarat province, southern Thailand (only known from the type locality).

####### Key to the troglomorphic *Coecobrya* of Thailand

This key includes *C.sirindhornae* sp. n. and all other species of Thailand which have long antennae (more than 2.5 times as long as the cephalic diagonal). All are cave restricted.

**Table:** 

1	Outer maxillary lobe with 3 sublobal hairs; a single chaeta m on labium	**2**
–	Outer maxillary lobe with 4 sublobal hairs; two chaetae m on labium; claw with unpaired inner tooth	**3**
2	Labial chaetae as mrel1l2; claw without unpaired inner tooth	***C.cavicta* Nilsai & Zhang, 2017**
–	Labial chaetae as mRel1l2, claw with unpaired inner tooth	***C.chumphonensis* Zhang & Nilsai, 2017**
3	Labial chaetae as M1m2rel1l2; antennae 5–7 times as long as cephalic diagonal; tita without a dorso-distal smooth chaeta in addition to the tenent hair; claw moderately slender	**4**
–	Labial chaetae as m1m2rel1l2, ; antennae >8 times as long as cephalic diagonal; tita with a dorso-distal smooth chaeta in addition to the tenent hair; claw very slender	***C.sirindhornae* sp. n.**
4	Postlabial chaeta X4 ciliate; mac a1 present on Abd. I	***C.polychaeta* Zhang & Nilsai, 2017**
–	Postlabial chaeta X4 smooth; mac a1 absent on Abd. I	**C.cf.polychaeta Zhang & Nilsai, 2017**

## Discussion

*Coecobrya* is, among Collembola, one of the genera that exhibit most frequently morphological modifications considered to be linked to subterranean environments (Christiansen and Bellinger 1992, Deharveng 1990, Jordana 2012, Nilsai et al. 2017). As illustrated on Fig. 1E‒G, its cave species show various degrees of troglomorphy. A first type corresponds to forms of small size, with short antennae and short appendages, resembling epigean species of the genus (Fig. 1E). In Thai caves they are mostly linked to guano deposits (Deharveng 1990). A second type displays troglomorphic characters, i.e., long antennae and large body size (Fig. 1F). *Coecobryasirindhornae* sp. n. described here belongs to this second type. Its troglomorphy is stronger than that of all other species of the genus, expressed as extremely long antennae, slender claw, and large body size (Fig. 1G). Like other described troglomorphic *Coecobrya* of Thailand (Nilsai et al. 2017), *C.sirindhornae* sp. n. is linked to oligotrophic habitat in the dark zone of the cave where it has been discovered, living in an atmosphere permanently wet and moist. All these species are very rare and narrow-range endemics in the country.

The first highly troglomorphic *Coecobrya*, *C.nupa*, was described by Christiansen and Bellinger in 1992 from Hawaii. Later on, Nilsai et al. (2017) described two long-antennae species, *C.chumphonensis* and *C.polychaeta* from Thailand. The antennae of *C.sirindhornae* sp. n. are distinctly longer. They are even longer than those of the most troglomorphic tropical Collembola, i.e., an undescribed Paronellidae from Laos (figured in Culver and Pipan 2009 where it is mistakenly cited from Cambodia) and an undescribed *Cyphoderopsis* from Sumatra (Deharveng and Bedos 2000), both with antennae approx. two times longer than (body + head). The elongation of antennae in *C.sirindhornae* sp. n. is only similar to that of *Verhoeffiellalongicornis* recently redescribed by Lukić et al. (2018) from the Dinarides in Europe, i.e., under temperate climate. The presence of such highly troglomorphic *Coecobrya* in Satun province is unexpected and raises evolutionary questions relative to the climatic drivers of colonisation, diversification and adaptation. Following the discovery of a rich troglobitic fauna in the lava tube fauna of Hawaii, Howarth (1973) was the first to challenge the view that cave adapted species were absent or exceptional in the tropics and proposed a bioclimatic model to account for this (Howarth 1980). This presence of true troglobites under tropical climate was later confirmed by Deharveng (1987) for Collembola. However, it was till recently admitted that tropical cave species rarely reach levels of troglomorphy as marked as some temperate species (Deharveng and Bedos 2000). The present discovery is a new compelling evidence that morphological modifications linked to cave life are often as strong in the lowland tropics than in temperate regions.

As Thailand is under tropical climate, cave temperature in the dark zone of lowland Thai peninsula is warm. It ranges from 23 to 30 degree Celsius (an average of 25‒26 degree Celsius for the caves studied so far) while humidity is approx. 70–93% (unpublished observations). The new highly troglomorphic species described here is restricted to a single small chamber (0.8 × 3‒4 m) without organic resources, where humidity is very high (> 90%), but temperature is only 23‒24 degree Celsius, a value very low in caves of the region. The karst where the cave is developed is a small outcrop of low elevation, that cannot account for the low temperature observed. The other troglomorphic species of southern Thailand were also often collected in relatively low temperature microhabitats. These rough ecological data suggest that highly troglomorphic Collembola may require a specific environment in tropical caves, not only oligotrophic habitats. This remains to be investigated in more detail.

Three of the four most troglomorphic *Coecobrya* known in Thailand (*C.sirindhornae* sp. n., *C.polychaeta*, and *C.cavicta*) are limited to Satun caves. The fourth one is *C.chumphonensis*, from the province of Chumphon, 380 km to the north. The karst of Satun highlights the region’s most complete Palaeozoic geological history and outstanding features of karst landscape that have developed during long geological periods (Department of Mineral Resources 2014). The limestone outcrop where the cave is developed is Ordovician of the Thung Song Group (480‒445 million years ago), one of the oldest thick sequence of carbonate rock in Thailand (Wongwanich et al. 1990). The last emergence of the limestones above sea level in the region, i.e., the oldest possible date of cave colonisation, is in Jurassic after the continental collision in the Late Triassic (Department of Mineral Resources 2014). This may have allowed Collembola to early colonise caves and to evolve in this habitat over millions of years. Molecular analyses like those initiated these last years (Nilsai et al. 2017, Zhang et al. 2018) will be useful to understand the origin and regional diversification of the *Coecobrya* lineage.

This exceptional richness in troglomorphic species of the karst of Satun highlights that the country’s first UNESCO Global Geopark (Satun Geopark) is also a spot of major biological importance. Regarding *C.sirindhornae* sp. n. itself, its area of occurrence is extremely reduced to a small chamber in a small cave of a small isolated hill. The hill is approx. 110 × 250 m and distant of approximately 400 m from the closest neighbouring limestone hill, which is approx. 500 × 2500 m The cave is occupied by a temple and surrounded by agricultural lands. Two caves of the same small hill were surveyed but did not provide any specimen of the new species. All this makes *C.sirindhornae* sp. n. highly vulnerable in the face of growing anthropic disturbance which are spreading over Thailand karsts, especially in lowland. The survey of neighbouring hill caves is on the way to better evaluate the fine distribution and vulnerability of this remarkable species. In a broader context, this discovery underlines a need higher than expected for rapid evaluation and assessment of the cave fauna of the numerous karstic outcrops spread on the plain of southern Thailand, and of the current threats affecting these karsts.

## Supplementary Material

XML Treatment for Coecobrya sirindhornae
